# Comprehensive analysis reveals PLK3 as a promising immune target and prognostic indicator in glioma

**DOI:** 10.32604/or.2024.050794

**Published:** 2025-01-16

**Authors:** TIANYUN ZHU, CUNYAN ZHAO, RUI GONG, AO QIAN, XIAOSHU WANG, FANGHUI LU, GANG HUO, LIANGJUN QIAO, SONG CHEN

**Affiliations:** 1 Department of Neurosurgery, The First Affiliated Hospital of Chongqing Medical University, Chongqing, 400016, China; 2 Institute of Life Sciences, Chongqing Medical University, Chongqing, 400016, China; 3 College of Basic Medicine, Chongqing Medical University, Chongqing, 400016, China

**Keywords:** Polo-like kinase 3 (PLK3), Glioma, Immune response, Prognosis, Immune microenvironment

## Abstract

**Background:**

PLK3, which played an important role in cell cycle progression and stress response, was identified as highly expressed in various carcinomas. However, the functions, molecular characteristics, and prognostic value of PLK3 in glioma remained unexplored.

**Methods:**

We analyzed PLK3 expression in glioma samples from multiple databases. Both overexpression and knockdown of Plk3 were performed to investigate tumor cell growth in glioma, and the transplanted glioma mouse model demonstrated the role of Plk3 on tumor progression. Immunohistochemistry was conducted to detect PLK3 expression and immune cell infiltration. The trans-well assay for PLK3 on the immune cells recruitment was also determined. Additionally, we further evaluated the correlation between PLK3 and PD-1/PD-L1 as well as other immune checkpoints.

**Results:**

We found that an increased level of PLK3 was associated with malignancy and poor prognosis of glioma, and further validated that PLK3 promoted glioma progression. PLK3 also played a crucial role in immune response and was involved in Tcell immune suppression. Specifically, we revealed that CD8^+^ and CD4^+^ Tcell infiltration was decreased in Plk3 overexpressed xenografts. Furthermore, it was predicted that PLK3 was synergistic with other checkpoint members in glioma. In general, high expression of PLK3 was associated with a malignant process and poor prognosis in glioma patients.

**Conclusion:**

Our findings indicated that PLK3 expression level was highly correlated to the malignancy of gliomas, and we validated that PLK3 could promote the GBM progress *in vitro* and *in vivo*. Furthermore, PLK3 played important roles in Tcell and neutrophil immune response in glioma. Besides, the conspicuous association between PLK3 and other immune checkpoints was also observed. Crucially, high-level PLK3 expression was revealed to be related to poor clinical prognosis. These results demonstrated that PLK3 may serve as a prognostic biomarker and a potential target for glioma.

## Introduction

Glioma is the most common malignant tumor in the central nervous system. Due to its infiltrative growth, it is difficult to completely resect the tumor by routine surgery [1].

Although comprehensive treatments, including total section, radiotherapy, and chemotherapy, are adopted, postoperative survival is still limited due to the high recurrence and malignant transformation rates of glioma, especially glioblastoma (GBM) [2]. Historically, according to histopathology, gliomas have been divided into low-grade gliomas (LGG) (World Health Organization [WHO] grades I and II) and high-grade gliomas (WHO grades III and IV) [3]. Considering the limitation of this classification, the WHO put forward a new classification by integrating tumor morphology, *IDH* status, and 1p19q co-deletion status in 2016. In recent years, with the development of molecular mechanism research, several genes have been used to evaluate the clinical features and prognosis of patients with glioma [4].

Polo-like kinase 3 (PLK3) is an immediate-early gene that plays an important role in cell cycle progression and the stress response [5,6]. One study reported that *IDH* mutations may affect the prognosis of patients with LGG by influencing PLK3 and related DNA damage repair pathways. Moreover, the sensitivity of temozolomide may be affected by PLK3 in LGG and GBM [7].

Previous reports have suggested that PLK3 is distinctively expressed in different carcinomas. Reduced PLK3 expression has been observed in colon, lung, liver, and kidney cancer [8–11], while PLK3 upregulation has been noted in ovarian and breast cancer, indicating a poor prognosis and short survival [12,13]. However, studies reporting PLK3 expression and function in glioma are scarce. To explore the status of PLK3 in glioma, RNA sequencing (RNA-seq) data of glioma samples were obtained from the Chinese Glioma Genome Atlas (CGGA) and The Cancer Genome Atlas (TCGA) datasets. As far as we know, this is the first integrative study to explore the characteristics of PLK3 at both the clinical and molecular levels in whole-grade glioma. Our analysis revealed a compelling correlation between elevated PLK3 expression and poor prognosis among patients with glioma. Furthermore, we demonstrated that PLK3 plays a key role in glioma development. Finally, we confirmed that PLK3 expression can remodel the glioma microenvironment and suppress T-cell immune function. Collectively, our results suggest that PLK3 has the potential to become a diagnostic marker and therapeutic target for glioma.

## Materials and Methods

### Cell culture

GL261-luc and U251 cell line were cultured in DMEM (Procell, PM150210) with 10% FBS (Vazyme, F101-1). Neutrophils and T-cells, isolated from C57BL6/J mouse, were cultured in 1640 (Procell, PM150110) with 10% FBS (Vazyme, F101-1). Cells were cultured in a humidified incubator at 37°C with 5% CO2 and atmospheric O2. Gl261-luc cell line was gifted from Yan lab, Nanjing University, Jiangsu, China. U251 cell line was Gifted from Shi lab, Third Military Medical University, Chongqing, China. Mycoplasma contamination was detected by PCR routinely, and cultured cells had no Mycoplasma contamination. Sequences are below:

Fwd 1. ACT CCT ACG GGA GGC AGC AGT A

Fwd 2. CCT AAA GGA ATT GAC GGG AAC CCG

Rev. TGC ACC ATC TGT CAC TCT GTT AAC CTC

### Glioma tissues collection

The area of glioma resection was the edge expansion of MRI-DWI image of 1–1.5 cm, including tumor and para-carcinoma tissues required in our experiment, which did not affect medical decision-making. In this study, we collected 6 pairs of tumor and para-carcinoma tissues per grade for WHO 2, WHO 3, and WHO 4 grade. All the patient tissues were collected in the First Affiliated Hospital of Chongqing Medical University following study protocol approval by The First Affiliated Hospital of Chongqing Medical University Institutional Review Board (Approval number: K2023-551). Using medical records and biological specimens obtained during previous clinical diagnoses and treatments, informed consent has been waived for this study. All tumors were subjected to pathological review and histological confirmation by an M.D. from neurosurgery before analysis.

### Mice modeling

The animal experiments were authorized by IACUC-CQMU. IACUC processing number: IACUC-CQMU-2024-01074. The GL261 luciferase-expressing (GL261-luc) cell line (Gifted from Yan Lab, Nanjing University, Jiangsu, China). 1 × 10^5^ GL261-luc were injected into the striatum (1.8, −0.4, −2.9) of C57BL/6J mice aged 8–10 weeks, 5 mice per group. *In vivo* imaging was taken after two weeks xenografts.

### Data collection

Four types of transcriptome data from patients diagnosed with glioma (WHO II–IV) were used: TCGA data (RNA-seq, n = 670) (http://cancergenome.nih.gov/); CGGA data (RNA-seq, n = 1018) (http://www.cgga.org.cn); the GSE16011 and GSE182109 database (n = 276) downloaded from Gene Expression Omnibus (GEO). The copy number variation profile (n = 667) and somatic mutation data (n = 654) from the TCGA data portal for the patients with corresponding RNA-seq data (http://cancergenome.nih.gov/).

### Bioinformatics analysis

GISTIC 2.0 was used to analyze the copy number alterations, which are associated with PLK3 expression. If an alternation peak was observed, the GISTIC analysis was used to obtain the threshold copy number at the alteration peak. Spearman’s correlation analysis was performed to acquire genes related to PLK3. Gene Ontology analysis in the R project was performed to detect the biological functions of related genes to draw a heatmap, and T-cell-specific gene sets were obtained from the AmiGO 2 Web portal. The inflammation-related metagenes and the calculation of metagene expression have been described in previous studies [14,15]. The packages used in this manuscript were as follows: clusterProfiler, corrgram, pheatmap, clusterProfiler, pheatmap, maftools, beeswarm, survival, ggplot2, circlize, corrplot.

### Plk3 short hairpin RNA

For Plk3 knockdown studies, GL261-luc cells were infected with lentiviral control short hairpin (sh)RNA or Plk3 shRNA, as previously validated. Non-targeting control (Sigma Aldrich, SHC016-1EA) and Plk3-specific shRNAs (Sigma Aldrich, TRCN0000027596 [GATGCTGACAACATATACATT], TRCN0000027671 [GCTGCATCAAGCAGGTTCATT], 5′ to 3′) were used in the pLKO.1 vector.

### Lentivirus production

Lentiviruses producing shRNAs against PLK3 messengers, as well as scrambled shRNA (Sigma-Aldrich, USA), were prepared using pMD2.G and psPAX2 plasmids according to a standard protocol. Briefly, HK293FT packaging cells were transfected with 45 μg polyethylenimine and incubated for 8 h before replacing the culture medium with 8 mL complete Dulbecco’s Modified Eagle Medium. The infectious conditioned medium (CM)-containing viruses were collected at 48- and 72-h following transfection.

### Quantitative polymerase chain reaction

Reverse transcription of extracted RNA was performed using Superscript II reverse transcriptase (Vazyme, China, cat: R233-01). For quantitative polymerase chain reaction (PCR), 10 μL reaction volume containing 2 μL complementary DNA (10 ng/μL), 0.4 μL of each forward and reverse target primer (10 μM), 5 μL Power SYBR Green mix (Vazyme, China, cat: Q711-02), and nuclease-free water up to 10 μL were used. Triplicate samples were prepared for each target gene.

PLK3 forward primer: TTTTCGCACCACTTTGAGGAC; PLK3 reverse primer: GAGGCCAGAAAGGATCTGCC. The GAPDH sequences are as follows: Forward primer-GGAGCGAGATCCCTCCAAAAT; Reverse primer-GGCTGTTGTCATACTTCTCATGG.

### Immunoblotting analysis

Tissues and cells were lysed in a splitting buffer (pH 7.4) containing a protease-inhibitor cocktail. The whole protein was separated by 10% sodium dodecyl sulfate-polyacrylamide gel electrophoresis and transferred to 0.45-μm polyvinylidene fluoride membranes. The membranes were subsequently incubated with antibodies against PLK3 (1:1000, Proteintech, USA, cat: 10977-1-ap) and GAPDH (1:10000, Proteintech, USA, cat: 60004-1-Ig) overnight at 4°C. After incubation, the membranes were washed three times with gentle agitation in TBST and incubated with secondary antibodies (1:5000, anti-mouse/anti-rabbit, Proteintech, China, SA00001-1/SA00001-2) for 1 h at room temperature.

### Cell viability assay

The effect of PLK3 knockdown/overexpression on the viability of GL261-luc cells and U251 (Gifted from Shi lab, Third Military Medical University, Chongqing, China) cells treated with GW843682X (Topscience, CN, CAS 660868-91-7) were determined using the Cell Counting Kit-8 (Life-ilab, China, cat: AC11L054) according to the manufacturer’s instructions.

### Transwell assays

Unstimulated migration was measured using a 3-μm pore Transwell system. CM from the PLK3 knockdown GL261-luc cell line was placed in the bottom of a 24-well plate. Educated neutrophils were added into the top chamber and incubated at 37°C in 5% carbon dioxide for 2 h. Images were then taken of the 24-well plate, and cell numbers were counted using ImageJ software.

### Immunohistochemical staining

Deparaffinized and dehydrated sections were boiled for 30 min in 1 × citrate buffer and incubated with endogenous peroxidase blockers for 10 min. The sections were then sequentially incubated with primary antibodies and secondary antibodies (AiFang Biological, China, cat: AFIHC001) and visualized with 1 × DAB (AiFang Biological, China, cat: AFIHC004). The sections were counterstained with haematoxylin, and representative images were captured under an inverted phase contrast microscope. Antibodies against the following targets were used: Ly6G (1:400, Biolegend, USA, cat: 127601), PLK3 (1:200 Proteintech, China, cat: 10977-1-ap), CD8 (1:200, BOSTER, China, cat: 98941), CD4 (1:200, BOSTER, China, cat: 0766R), PD-1 (1:400, CST, USA, 86163), and PD-L1 (1:400, CST, USA, 13684). The number of CD8^+^/CD4^+^ T-cells and neutrophils was assessed by ImageJ software.

### Statistical analysis

Differences in variables between groups were evaluated by the student’s *t*-test, one-way analysis of variance, or Pearson’s chi-square test. The prognostic value of PLK3 was estimated by the Kaplan–Meier analysis and the Cox proportional hazards model using the R project. Other statistical computations and figure drawing were performed using several packages (ggplot2, pheatmap, pROC, and corrgram) of the R project, version 4.1.0 (http://www.r-project.org). For all statistical methods, *p* < 0.05 was considered statistically significant.

## Results

### PLK3 was associated with molecular and clinical malignancy characteristics in glioma

**Figure 1:**
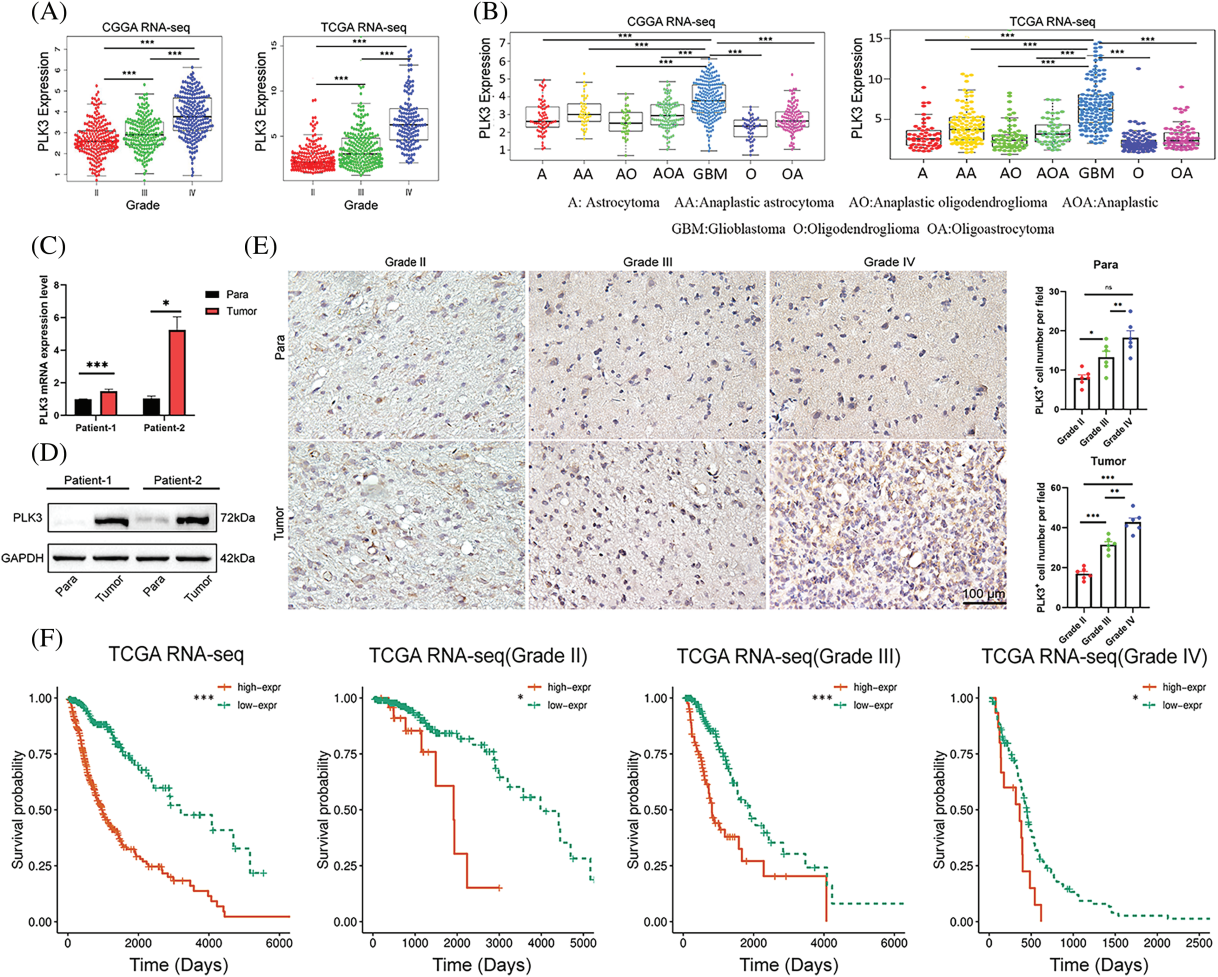
Clinical parameters in association with PLK3 expression. (A) PLK3 expression increased by WHO grade. (B) PLK3 was enriched in GBM. (C) qPCR and (D) western blotting analysis for PLK3 expression in the tumor or para-carcinoma tissues. (E) Immunohistochemical analysis of PLK3 expression in tumor or para-carcinoma tissues by WHO grade. Scale bar: 100 μm. (F) Kaplan–Meier survival analysis of patients with whole-grade gliomas and gliomas of grades II, III, and IV in TCGA dataset. Data are presented as the mean ± s.e.m. In our analysis, ns, *, **, and *** indicate no significant difference, *p < 0.05, **p < 0.01, and ***p < 0.001, respectively.

With the RNA-seq analysis of glioma, the expression of PLK3 increased with glioma grade, and it was highest for WHO grade IV glioma (Fig. 1A; Fig. S1A). Compared with other subtypes, such as astrocytoma, oligodendroglioma, and oligoastrocytoma, as well as their anaplastic variations, GBM was related to higher PLK3 expression (Fig. 1B; Fig. S1B,C). To validate these findings, real-time PCR and Western blotting were performed to detect PLK3 expression in human samples. The results showed increased expression of PLK3 in samples from patients with glioma (Figs. 1C,D; Fig. S1D). Furthermore, immunohistochemistry was performed to evaluate the expression of PLK3 in these samples. The results revealed a marked increase in PLK3 expression in glioma tissue (Fig. 1E). The survival assay showed a poor prognosis in PLK3 expression in WHO whole-grade gliomas (Fig. 1F; Fig. S1E). As an essential indicator of prognosis, gliomas with *IDH* wild-type status had considerably higher expression of PLK3 than gliomas with *IDH* mutations (Fig. S1F). Thus, PLK3 expression was positively associated with the malignance and aggressiveness of glioma.

Molecular classification provides a new method to predict the outcomes of different patients with glioma. Classical, mesenchymal, neural, and proneural are the molecular subtypes of glioma according to the definitions of TCGA and CGGA networks. As shown in Fig. S1G, a significant difference was found in the distribution of PLK3 expression among the four subtypes. Compared with other subtypes, the highest PLK3 expression was detected in the mesenchymal subtype, which showed the worst prognosis for patients [16]. Moreover, we analyzed scRNA-seq data from 44 fragments of tumor tissue (GSE182109) [17], and found PLK3 was highly expressed in MES cells, which was consistent with the analysis in pathology subtypes of GBM (Fig. S1H). Given the intimate relationship with glioma malignance, PLK3 may play an important role in glioma progression. Meanwhile, PLK3 may be considered a biomarker of the mesenchymal subtype.

### PLK3 expression correlated with distinct genomic alteration patterns

**Figure 2:**
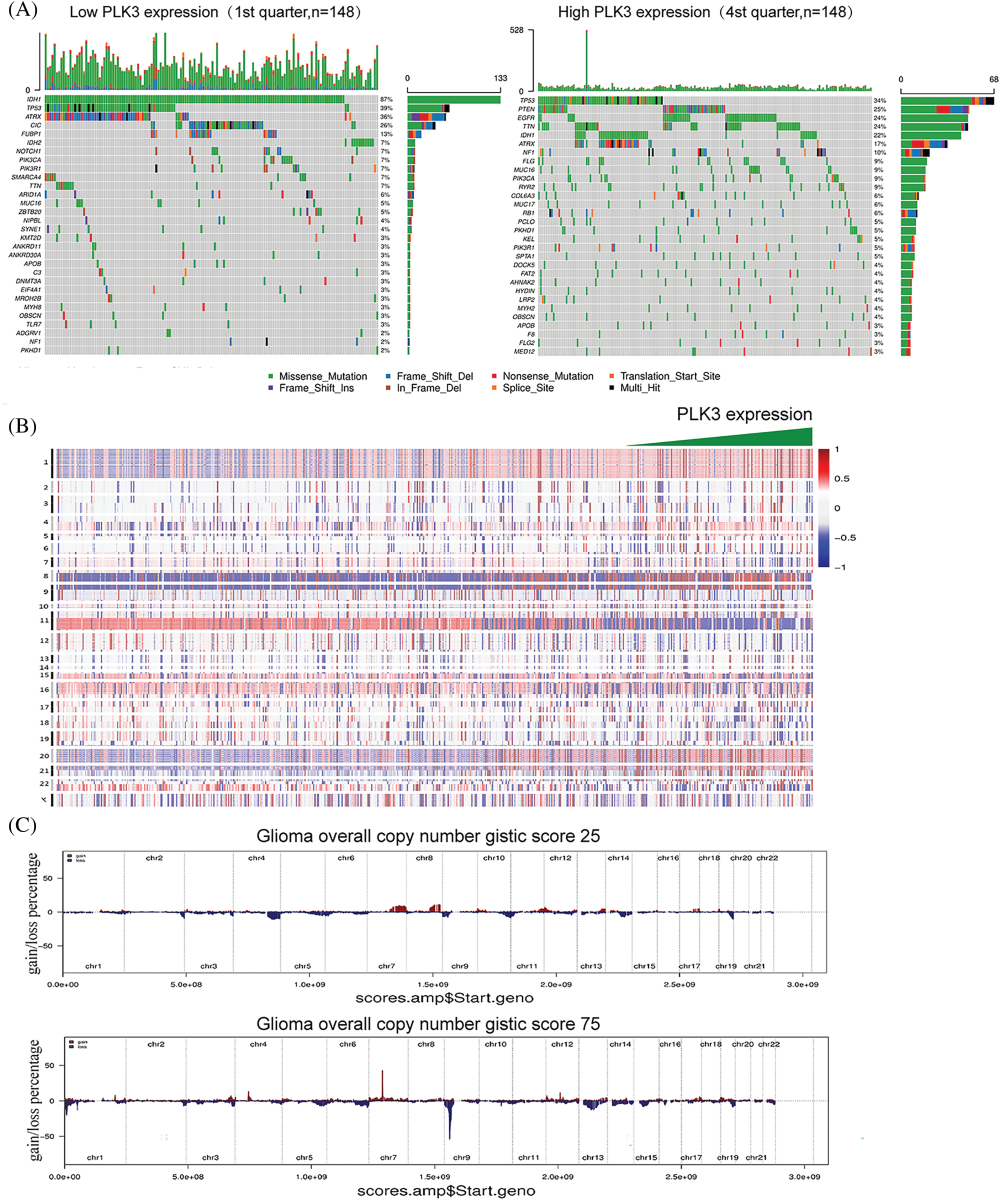
Genomic profiles associated with different PLK3 expression levels. (A) Different somatic mutations were found in the low and high PLK3 expression groups of glioma. (B) The overall CNA profile with the increase in PLK3 expression. (C) GISTIC 2.0 amplifications and deletions in the different PLK3 expression groups of glioma.

Genetic mutation is a pervasive molecular mechanism underpinning glioma. Therefore, TCGA and the CGGA databases were analyzed to explore the characteristics of somatic mutations and copy number alterations in glioma. Parallel analyses were performed in two groups, three groups, or four groups classified in ascending order of PLK3 expression. Low and high PLK3 expression groups were formed to compare the mutation frequency. More somatic mutations were revealed in cases with high PLK3 expression (1st *vs*. 2nd half: 6938 *vs*. 31,404 mutations; 1st *vs*. 3rd tertile: 4104 *vs*. 7,446 mutations; 1st *vs*. 4th quartile: 3018 *vs*. 5833 mutations). In the low PLK3 expression group, mutations in *IDH1*, *CIC*, and *FUBP1* were significantly detected. In the high PLK3 expression group, mutations in *TTN*, *EGFR*, *ATRX*, and *PTEN* were enriched (Fig. 2A; Fig. S2A). Furthermore, the high PLK3 expression group frequently showed amplification of chromosome 7 and deletion of chromosome 10, both of which were typical genomic events in GBM (Fig. 2B). In the high PLK3 expression samples, frequently amplified genomic regions containing oncogenic driver genes, such as *EGFR* (7p11.2), *PDGFRA* (4q12), *CDK4* (12q14.1), and *CCND2* (12p13.32), were accompanied by *CDKN2A* and *CDKN2C* (9p21.3 and 1p32.3) deletion peaks (Fig. 2C; Fig. S2B).

### PLK3 participated in multiple malignant biological processes

**Figure 3:**
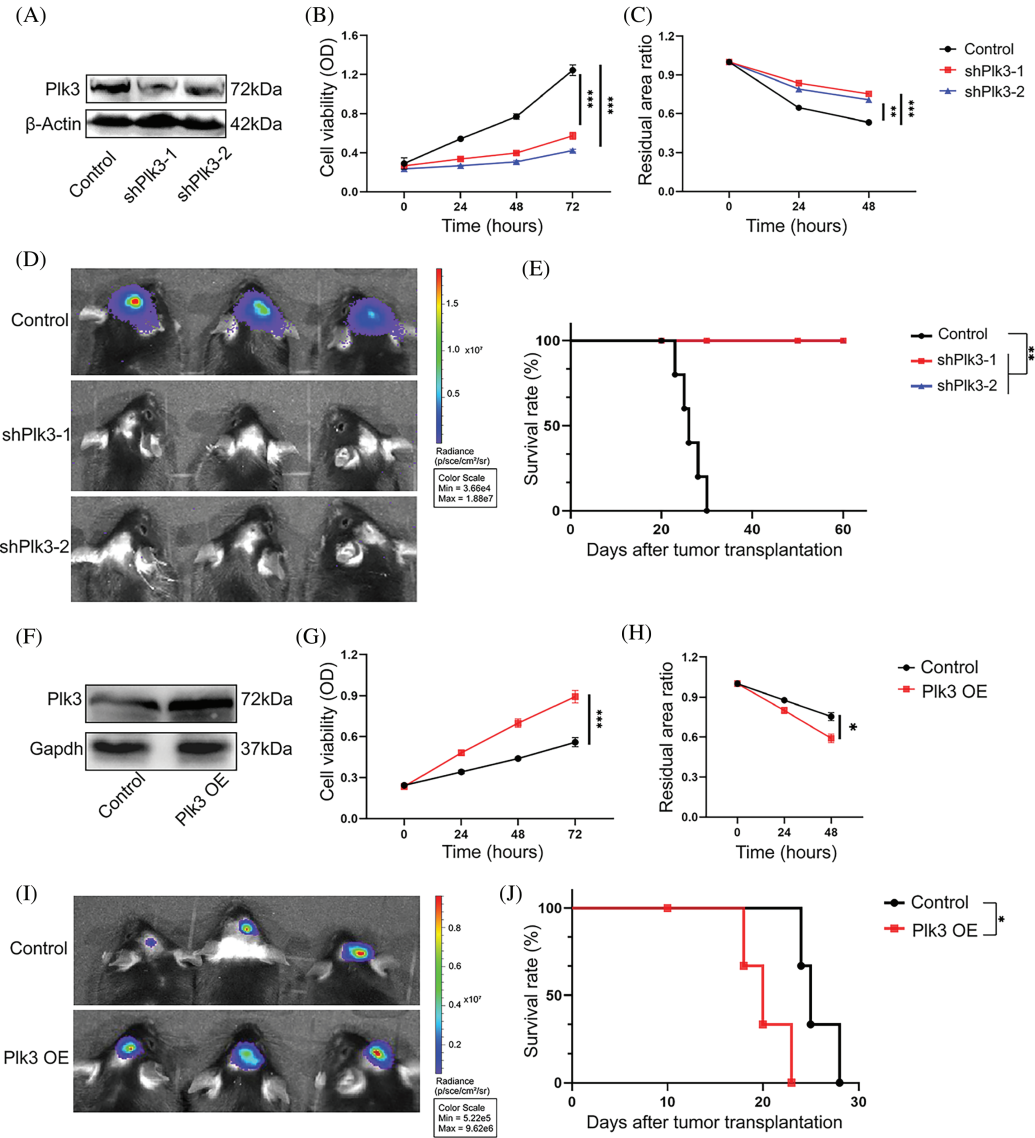
PLK3 regulated GBM growth. (A) Western blotting analysis showed that PLK3 was knocked down in murine GL261-luc tumor cells. Cell viability was measured by the (B) CCK-8 assay and the (C) wound healing assay of *Plk3* knockdown cells compared with control cells. (D) Representative bioluminescence images of tumors in the control and *Plk3* knockdown cells (14 days). (E) Survival of the control group compared with the *Plk3* knockdown group (n = 5 mice per group). (F) Western blotting analysis of the vector or overexpressed cells in murine GL261-luc tumor cells. Cell viability was measured by the (G) CCK-8 assay and the (H) wound healing assay of *Plk3*-overexpressing cells compared with control cells. (I) Representative bioluminescence images of tumors in the control and *Plk3* knockdown cells (14 days). (J) Survival of the vector compared with the *Plk3*-overexpressing group (n = 5 mice per group). *p < 0.05, **p < 0.01, and ***p < 0.001, respectively.

To validate the functions of PLK3 in glioma, we genetically knocked down Plk3 in the GL261 cell line by lentiviral infection (Fig. 3A; Fig. S3A). Plk3 knockdown cells showed reduced growth compared with their controls, as well as reduced migration (Fig. 3B,C; Fig. S3B,C). To confirm these results, Plk3 knockdown GL261-luc cells were transplanted into the striatum of C57BL/6J mice. Fourteen days after transplantation, the animals were subjected to *in vivo* imaging. The results showed an improvement in survival in the mouse GBM model with Plk3 knockdown (Fig. 3D,E). To further investigate the role of PLK3 in GBM growth, we overexpressed Plk3 in the GL261 cell line by lentiviral infection (Fig. 3F; Fig. S3D). Overexpressing cells showed increased cell viability and migration ability (Fig. 3F; Fig. S3D). *In vivo*, the overexpressing group revealed poor survival (Fig. 3I,J; Fig. S3E,F).

Overexpressing cells showed increased cell viability and we performed the same experiments in the U251 cell line with the PLK3 inhibitor GW843682X at 8 μM (Fig. S4A,B) [18]. Consistently, we observed suppression of cell growth (Fig. S4C,D). Overall, these results demonstrate that PLK3 promotes GBM growth, and silencing the PLK3 gene strongly reduces viability, migration, and tumor-forming potential.

### PLK3 modulated the glioma immune microenvironment

**Figure 4:**
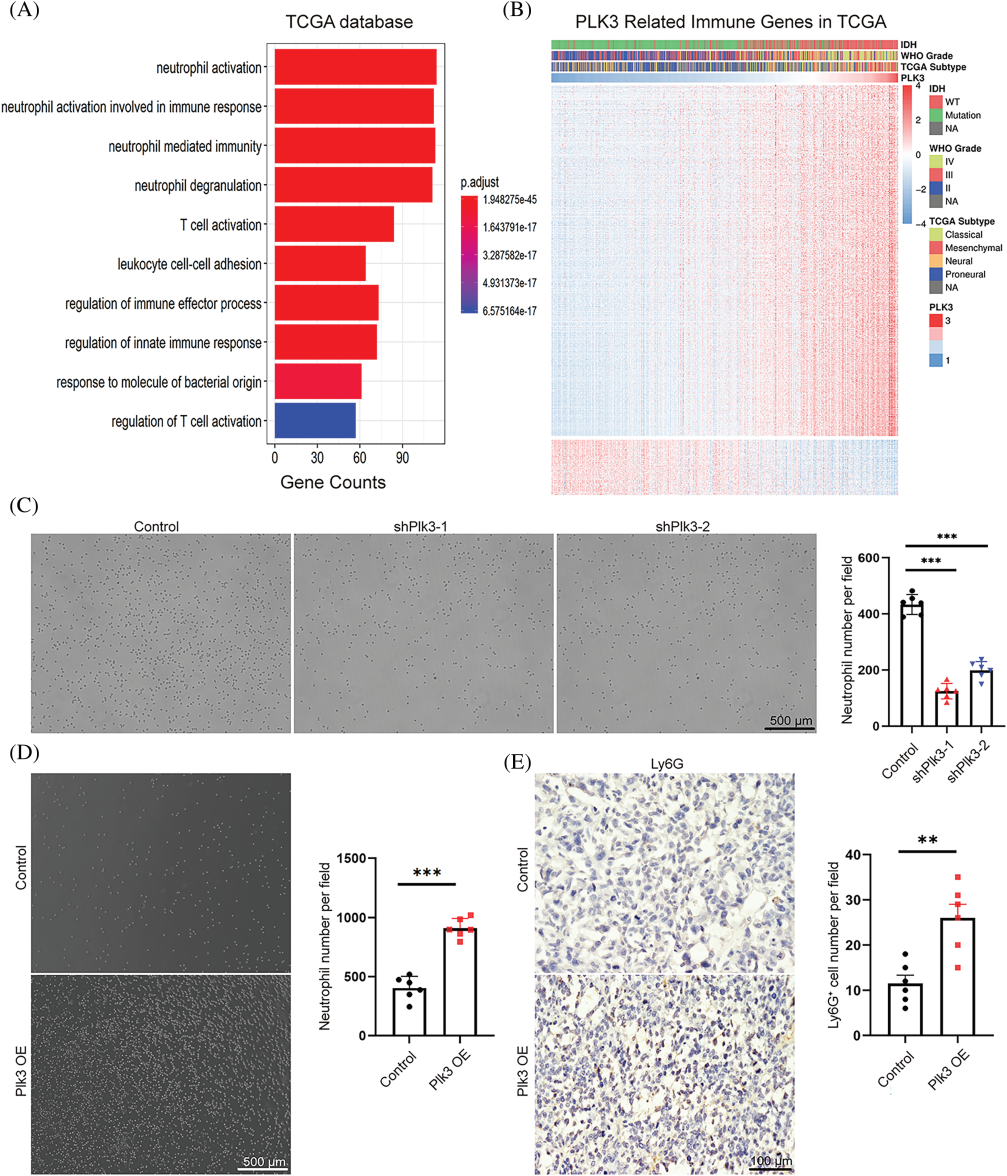
PLK3 expression was correlated with the immune response. (A) The Gene Ontology analysis demonstrated that PLK3 was mostly associated with the immune response in the TCGA dataset. (B) The heatmap analysis demonstrated that PLK3 was positively correlated with most immune genes in the TCGA dataset. (C) The transwell assay and statistic of neutrophil treated with control and *Plk3* knockdown tumor CM for 3 h. Scale bar: 500 μm. (D) Transwell assay and statistic of neutrophil treated with vector and *Plk3*-overexpressing tumor CM for 3 h. Scale bar: 500 μm. (E) Immunohistochemical staining and quantification of neutrophils (wild-type: n = 5; overexpressing: n = 5) in transplantation tumor tissues. Scale bar: 100 μm. ns, **, and *** indicate no significant difference, **p < 0.01, and ***p < 0.001, respectively.

Previous studies have reported that PLK3 plays an essential role in biological functions in both physiological and pathological states. However, the impact of PLK3 in the glioma microenvironment remains largely elusive. Hence, 1218 genes from the TCGA dataset and 2455 genes from the CGGA dataset that were strongly correlated with PLK3 according to Pearson correlation analysis (Pearson’s |R| > 0.5) were selected for the GO analysis to analyze their biological functions. In the TCGA dataset, genes that were correlated with PLK3 were mostly related to the inflammatory and immune responses (Fig. 4A). Similar results were observed in the CGGA dataset (Fig. S5A). In both the TCGA cohort and the CGGA cohort, normal biological processes, such as chemical synaptic transmission, were more commonly functions of negatively correlated genes (Fig. S5B,C).

Immune-response-related genes from the AmiGO 2 Web portal were sought to explore the correlation between PLK3 expression and the immune response [19]. Overall, 990 genes from the TCGA dataset and 872 genes from the CGGA dataset, all of which were mostly related to PLK3 (Pearson’s |R| > 0.4), were selected to draw the heatmaps. In the TCGA dataset, 855 genes were positively associated with PLK3 expression, while 135 genes were negatively associated with PLK3 expression (Fig. 4B). In the CGGA dataset, 864 genes were positively associated with PLK3 expression, while 8 genes were negatively associated with PLK3 expression (Fig. S5D). Accordingly, in glioma, the most relevant immune responses were positively associated with PLK3 expression. In particular, PLK3 expression was strongly correlated with neutrophil and T-cell immune activity. Therefore, we suggest that PLK3 may be involved in suppressed immune microenvironment remodeling.

Our previous analysis revealed a significant correlation between PLK3 expression and the neutrophil immune response in glioma (Fig. 4A). To validate these results, we gathered CM from PLK3 knockdown and overexpressing GL261 cells to detect naïve neutrophil chemotaxis. We observed a decrease in neutrophil migration in PLK3-deficient tumor CM (Fig. 4C) and an increase in neutrophil migration in CM from PLK3-overexpressing cells (Fig. 4D), indicating that PLK3 may promote neutrophil migration in the GBM microenvironment. Immunohistochemistry also showed higher neutrophil infiltration in the PLK3-overexpressing transplant group (Fig. 4E). These results suggest that PLK3 expression can affect neutrophil infiltration in GBM.

### PLK3 was involved in T-cell immune suppression in glioma

**Figure 5:**
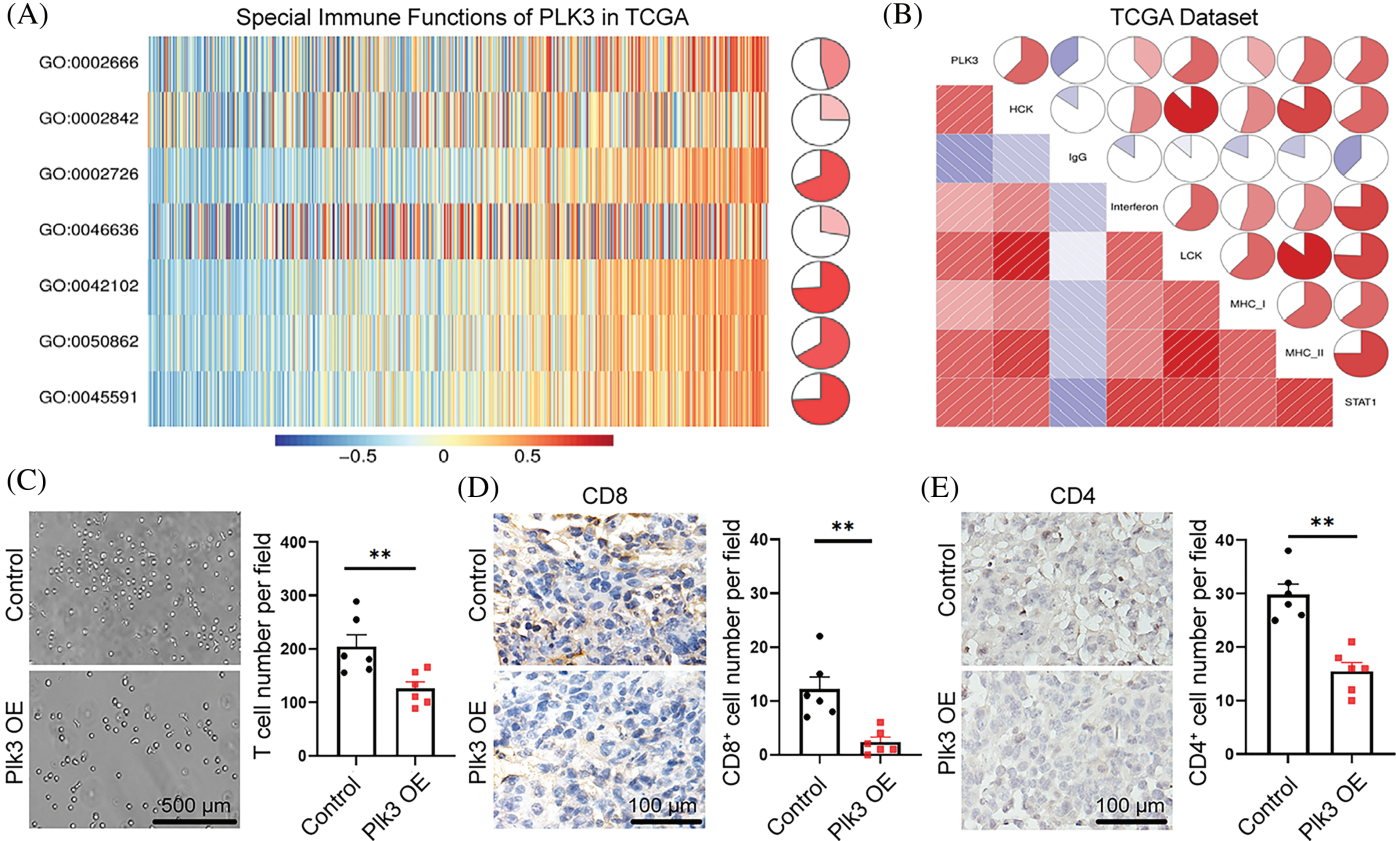
PLK3-related T-cell immunity in glioma. (A) PLK3-related T-cell immunity. GO: 0002666, positive regulation of T-cell tolerance induction. GO: 0002842, positive regulation of T-cell-mediated immune response to tumor cells. GO: 0002726, GO positive regulation of T-cell cytokine production. GO: 0046636, go negative regulation of alpha-beta T-cell activation. GO: 0042102, go positive regulation of T-cell proliferation. GO: 0050862, go positive regulation of T-cell receptor signaling pathway. GO: 0045591, go positive regulation of regulatory T-cell differentiation. (B) Relationship between the correlogram of PLK3 and inflammatory activity. Red indicates positive correlations and blue indicates negative correlations. (C) Transwell assay and statistic of CD8^+^ T-cells treated with vector and *Plk3*-overexpressing tumor CM for 3 h. Scale bar: 500 μm. (D and E) Immunohistochemical staining and quantification of CD4^+^ and CD8^+^ T-cells (wild-type: n = 6; overexpressing: n = 6) in transplantation tumor tissues. Scale bar: 100 μm. ns indicates no significant difference, **p < 0.01.

As there was a close association between PLK3 expression and T-cell immune response in glioma, the Gene Set Variation Analysis was performed to explore the connection between PLK3 expression and T-cell immunity in glioma. In TCGAc databases, an association between PLK3 expression and T-cell immunity was found in the form of positive regulation of T-cell tolerance induction, T-cell-mediated immune response to tumor cells, and T-cell cytokine production. We also found that PLK3 expression was negatively associated with the regulation of alpha-beta T-cell activation (Fig. 5A). These results indicated that PLK3 inhibited T-cell immunity in glioma. In the TCGA dataset, the expression of PLK3 was positively associated with HCK, interferon, LCK, MHC-1, MHC-2, and STAT-1, which are closely associated with macrophage activation, cell signal transduction, and antigen presentation. A negative correlation with immunoglobulin G, which represents the activity of B lymphocytes, was also detected (Fig. 5B). Accordingly, we consider that the function of PLK3 in the immune response in glioma is crucial.

To validate our analysis, we gathered the supernatant from PLK3-overexpressing cells to detect T-cell migration. The results showed that T-cell migration decreased (Fig. 5C). Moreover, CD4^+^ and CD8^+^ T-cells decreased in the Plk3-overexpressing tumor model (Fig. 5D,E). All of these results confirm that T-cell immune functions were suppressed in the high PLK3 expression group.

### PLK3 was highly correlated with immune checkpoint members and predicted immunotherapeutic efficacy

**Figure 6:**
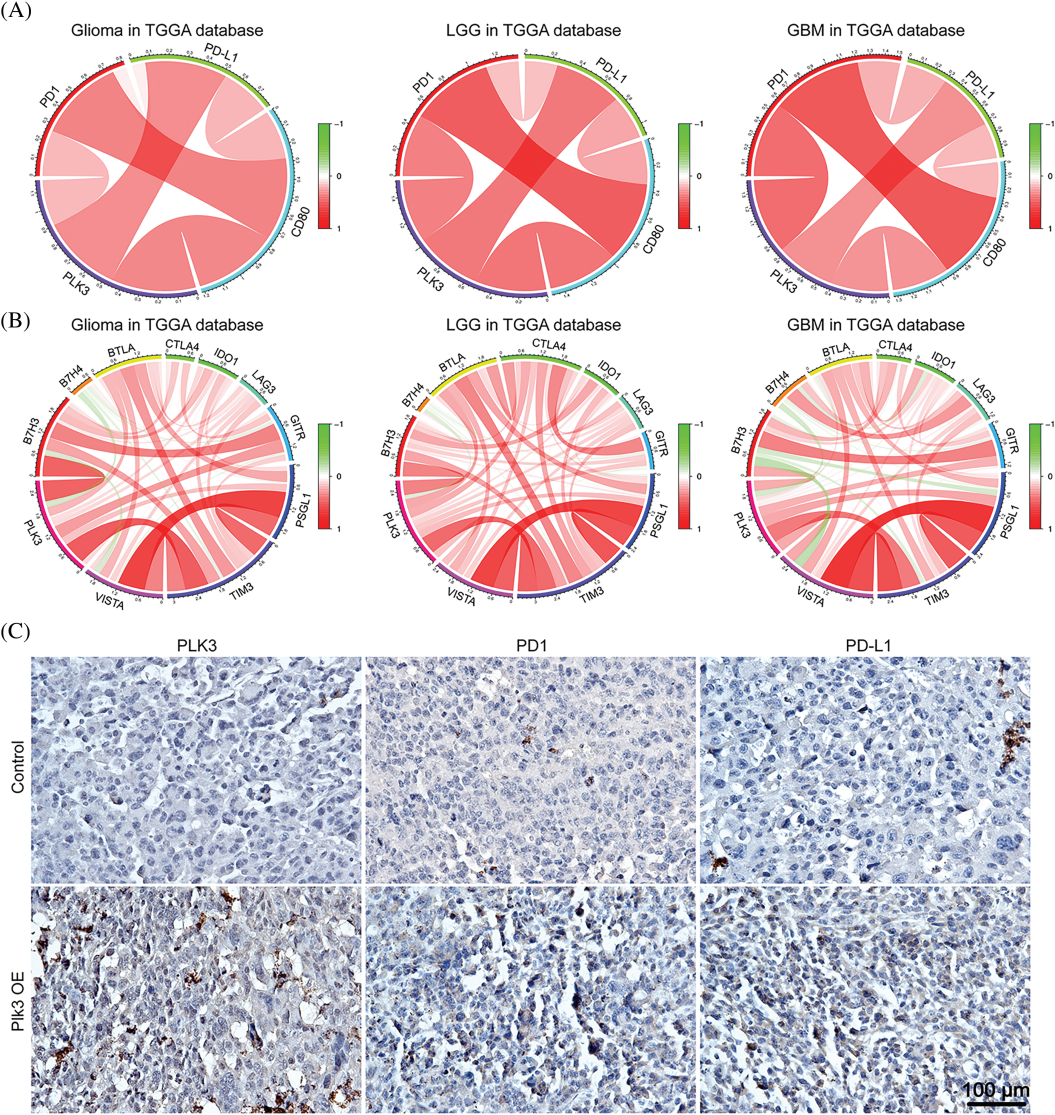
Relationships between PLK3 and immune checkpoints. (A) Correlations among PLK3, PD-1, PD-L1, and CD80 in whole-grade glioma, low-grade glioma, and GBM. (B) Associations between PLK3 and other immune checkpoints. (C) Immunohistochemical analysis of PLK3, PD-1, and PD-L1 in control and *Plk3*-overexpressing mouse tumors. Scale bar: 100 μm.

PD-1 and its receptor (PD-L1) are crucial immune checkpoints in tumors, and the binding of PD-L1 and PD-1 restrains the anti-tumor function of T-cell(s) and enhances regulatory T-cell activation, which enables tumors to achieve immune evasion [20,21]. Further, PD-L1 interacts with CD80 on the surface of activated CD8^+^ T-cells to suppress its anti-tumor activity [22]. Our previous results indicated that T-cells may be suppressed by immune checkpoints in GBM with high PLK3 expression. Therefore, Pearson’s correlation analysis was performed with PLK3, PD-L1, PD-1, and CD80 expression, both in the TCGA dataset and the CGGA dataset. The results showed connections between PLK3 and PD-1, and between PD-L1 and CD80, which revealed strong correlations among PD-1, PD-L1, and CD80 in whole-grade glioma, LGG, and GBM (Fig. 6A; Fig. S6A). To further explore the relationship between PLK3 expression and immune checkpoints, we chose several immune checkpoint genes to explore the relationship between PLK3 expression and glioma, including *VISTA*, *TIM3*, *PSGL1*, *LAG3*, *IDO1*, *CTLA4*, *BTLA*, *B7H4*, and *B7H3*. The results showed that PLK3 expression was tightly correlated with *TIM3* and *B7H3* in whole-grade glioma, LGG, and GBM (Fig. 6B; Fig. S6B).

Moreover, immunohistochemistry of PD-1 and PD-L1 revealed an increasing positive signal in Plk3-overexpressing tumors (Fig. 6C; Fig. S6C). Thus, we believe that PLK3 may play an important role in the regulation of the PD-1/PD-L1 pathway. All these results revealed that PLK3 is correlated with immune checkpoints, which may be reasonable for T-cell immune suppression.

## Discussion

As the most common primary tumor of the brain parenchyma, the treatment of glioma has always been challenging. Although progress has been made in conventional treatment regimens, outcomes and prognosis are still poor due to the tendency of gliomas to infiltrate into the surrounding parenchyma [22]. Immunotherapy has demonstrated certain benefits in cancer treatment. The combination of immune checkpoint inhibitors has extended the survival of some patients with melanomas and non-small-cell lung cancer [23,24]. However, the efficacy of immune checkpoint inhibitors is limited and unpredictable in glioma, particularly GBM [25]. Recently, Pang et al. suggested that the stress response and double-strand break repair, in which PLK3 is involved, might play important roles in modulating the therapeutic response of glioma [7]. However, the status of PLK3 in glioma still remains unclear. Determining the molecular characteristics of PLK3 may help to define it as a new target for glioma therapy.

IDH is one of the most essential catalytic enzymes in the tricarboxylic acid cycle that catalyzes the oxidative decarboxylation of isocitrate to 2-oxoglutarate [7]. In earlier reports, *IDH* mutation status was regarded as a significant predictor of prognosis in patients with glioma, and it was associated with a better prognosis and longer survival than wild-type *IDH* [7,26]. Moreover, the mesenchymal subtype of glioma is associated with a high frequency of *NF1* abnormalities, which are associated with immunosuppression and aggression [16,22]. Crucially, compared with other subtypes, patients with the mesenchymal subtype of glioma have the worst prognosis, irrespective of whether the tumor is primary or recurrent [16].

In this study, we found that increased expression of PLK3 was associated with more malignant glioma in terms of higher WHO grade through multiple analyses, *IDH* wild-type status, and the mesenchymal subtype of glioma samples. *In vitro* experiments also confirmed that PLK3 expression promoted GBM growth in mice. Combining these results, high PLK3 expression showed a correlation with malignant entities. According to the analysis of somatic mutations in the different PLK3 expression groups, we found that mutations in *TTN*, *EGFR*, *PTEN*, and *NF1*, all of which were associated with high PLK3 expression, indicated a poor prognosis [16,27–29]. On the contrary, mutations in *IDH1*, *CIC*, and *FUBP1*, which were significantly higher in the low PLK3 expression group, were correlated with longer overall survival [30,31]. In the low PLK3 expression group, a high rate of 1p/19q co-deletion was observed. *FUBP1* and *CIC* are located on 1p and 19q, respectively, which might be the reason for the high level of mutations in these two genes in the low PLK3 expression group [31]. In contrast, a high level of chromosome 7 amplification with chromosome 10 deletion, which was a molecular characteristic of wild-type *IDH* GBM, was observed in the high PLK3 expression group [32]. For somatic copy number alterations, cases with high PLK3 expression not only demonstrated significant amplification peaks for *PIK3C2B*, *PDGFRA*, *EGFR*, and *CDK4*, which are defined as oncogenic drivers [33,34], but they also showed deletion peaks for tumor-suppressor genes, such as *CDKN2A* and *CDKN2C* [35]. Thus, we believe that PLK3 expression may play an important role in malignant biological processes. Clarifying the mechanism of PLK3 in gliomas may become a crucial step to cure this disease.

Recently, Vaughan et al. reported that PLK3 phosphorylated S20 of mutant p53 and affected cell growth, tumorigenicity, and invasion in various tumor cells [36]. However, siPLK3 can also rescue the GBM cell cycle suppressed by TUBA1A inhibition [37]. Our results confirmed that PLK3 can directly regulate glioma growth *in vivo* and *in vitro*.

Neutrophils, a key component of the glioma microenvironment, exhibit significant heterogeneity in function and phenotype [38,39]. Our research identified a strong correlation between PLK3 expression and the neutrophil immune response, along with evidence that PLK3 overexpression can promote neutrophil recruitment. Therefore, the role of PLK3 on neutrophils in glioma deserves further investigation. The relationship between PLK3 and T-cell immunity has not been studied before. In our analysis of the immune function of PLK3 in gliomas, a positive association between PLK3 and T-cell immunity was found in regulatory T-cell differentiation and T-cell tolerance induction, both of which imply T-cell anergy and glioma aggressiveness [40]. Furthermore, negative regulation of CD4^+^ and CD8^+^ T-cell activation was also detected. Negative regulation of CD4^+^/CD8^+^ T-cells may restrain T-cell immunity in glioma. *In vivo* immunohistochemistry and *in vitro* co-culture experiments showed that T-cell immune functions were suppressed. We found that PLK3 has close connections with PD-1, PD-L1, and CD80; therefore, it is possible that suppression of T-cell immunity by PLK3 may be attributed to the regulation of PD-1, PD-L1, or CD80 through PLK3. Nevertheless, these findings were correlative, and further studies that elucidate the relationship between PLK3 and T-cell function are essential.

Immune checkpoint inhibition, which has achieved success in treating the recurrence of a variety of cancer types, is an attractive therapeutic option [25]. Despite it being a vital immune checkpoint, few reports have evaluated the correlation between PLK3 and other immune checkpoints. PD-1, PD-L1, and CD80 are negative regulators of anti-tumor immunity that suppress the anti-tumor function of T-cells. Recently, a meta-analysis suggested that high *PD-L1* expression indicates poor overall survival in patients with glioma [21]. Liu et al. reported that high *PD-1* expression is associated with malignancy and glioma invasiveness [41]. In this study, we first revealed the tight correlation between PLK3 and *PD-1/PD-L1*, as well as *CD80*. Furthermore, we analyzed the relationship among PLK3 and several immune checkpoint genes. In both the CGGA dataset and TCGA dataset, PLK3 played a suppressive role in the anti-tumor immune response, and patients with high PLK3 expression in gliomas had a poor prognosis. Moreover, both *B7H3* and *TIM3* are positively correlated with high WHO grade and glioma aggressiveness [33,42,43]. As desirable correlations between PLK3 expression and some immune checkpoints were obtained, immune treatment combined with PLK3 inhibitors may be a novel approach to glioma therapy. Studies elucidating the immunoregulatory role of PLK3 in the glioma microenvironment and demonstrating that anti-PLK3 can prevent growth and promote immune-responsive gliomas are essential in the future.

In terms of prognosis, the present study unveiled that high PLK3 expression indicated shorter overall survival in patients with different WHO grades of glioma and GBM. Therefore, the immunotherapy restraining activity of PLK3 may improve the prognosis of patients with glioma.

## Conclusion

Through the analysis of transcriptomic and genomic profiling data, we found that PLK3 expression level was highly correlated to the malignancy of gliomas, and we validated that PLK3 could promote the GBM process *in vitro* and *in vivo* experiments. Furthermore, PLK3 played important roles in T-cell and neutrophil immune response in glioma. As well, the conspicuous association between PLK3 and other immune checkpoints was also observed. Crucially, high-level PLK3 expression was revealed to be related to poor clinical prognosis. These results demonstrated that PLK3 may serve as a prognostic biomarker and a potential target for glioma.

## Supplementary Materials

Supplementary Fig. S1 Survival Correlation and PLK3 expression glioma. (A) PLK3 expression increased by WHO grade in CGGA. (B) PLK3 was highly expressed in GBM. (C) GBM PLK3 expression level in GEPIA. (D) Quantification of PLK3 blot. (E) Kaplan–Meier survival analysis for patients with gliomas in whole grades, and gliomas in grade II, grade III, grade IV in CGGA dataset. (F) PLK3 highly expressed in IDH wild-type glioma. (G) PLK3 highly expressed in Mesenchymal subtype of glioma. (H) Analysis of scRNA-seq (GSE182109) conducted by Seurat 4.0. In our cohort, ns, * and *** indicated no significant difference, *p < 0.05, ***p<0.001, respectively.

Supplementary Fig. S2 Distinct somatic mutation and CNAs profiles associated with PLK3 expression. (A) Different somatic mutations were found in low and high PLK3 expression in glioma (1st tertile vs 3rd tertile, and 1st half vs 2nd half). (B). GISTIC 2.0 amplifications and deletions in different PLK3 expression in glioma (glioma overall copy number gistic score 33, 67, 50u and 50d).

Supplementary Fig. S3 (A) Quantification of PLK3 knockdown blots. (B) Cell status before added CCK8 in Plk3 knockdown cells. (C) Wounding healing assay for GL261 of Plk3 knockdown cells for 0h, 24h and 48h. Scale bars, 500μm. (D) Quantification of PLK3 overexpressed blots. (E) Cell status before added CCK8 in Plk3 knockdown cells. Scale bars, 200μm. (F) Wounding healing assay for GL261 of Plk3 overexpressed cells for 0h, 24h and 48h. Scale bars, 500μm. Blots quantified by ImageJ. Data are presented as the mean ± s.e.m. In our analysis, ns, **, and *** indicate no significant difference, **p < 0.01, and ***p < 0.001, respectively.

Supplementary Fig. S4 (A) Western blot and (B) qPCR to detect PLK3 inhibition in U251 treated with GW843682X. Inhibitor concentration was at 8 μM. (C) Cell viability was measured by CCK-8 assay. (D) Wounding healing of U251 treated with GW843682X. Scale bars, 500μm. Data are presented as the mean ± s.e.m. In our analysis, ns, *** indicate no significant difference, ***p < 0.001.

Supplementary Fig. S5 Gene ontology analysis for PLK3 in glioma. (A) and (B) Gene ontology analysis demonstrated that PLK3 was mostly associated with inflammatory response and immune response in CGGA datasets. Heatmap analysis demonstrated that PLK3 was positively correlated with most immune genes in CGGA datasets. (C) and (D) Gene ontology analysis demonstrated that PLK3 was negatively associated with biological process in CGGA and TCGA datasets.

Supplementary Fig. S6 (A) Correlations among PLK3, PD-1, PD-L1 and CD80 in whole gliomas, low-grade gliomas and GBM in CGGA. (B) Associations between PLK3 and other immune checkpoints in CGGA. (C) Statistic of PLK3/PD1/PD-L1 positive cell per field in Graphpad 8.0. Data are presented as the mean ± s.e.m. In our analysis, ns, * and *** indicate no significant difference, *p < 0.05, ***p < 0.001, respectively.

## Data Availability

The datasets presented in this study can be found here: http://cancergenome.nih.gov/ and http://www.cgga.org.cn.

## Abbreviations

PLK3: Polo-like kinase 3
PD-1: Programmed cell death protein 1
CGGA: Chinese Glioma Genome Atlas
TCGA: The Cancer Genome Atlas
GBM: Glioblastoma
LGG: Low-grade glioma
WHO: World Health Organization

## Supplementary Materials

The supplementary material is available online at https://doi.org/10.32604/or.2024.050794.
