# Supplementary figures and images for: Comprehensive analysis reveals PLK3 as a promising immune target and prognostic indicator in glioma
Source: Oncol Res. 2025 Jan 16;33(2):431–42. doi: 10.32604/or.2024.050794 (PMC11753997; doi:10.32604/or.2024.050794)

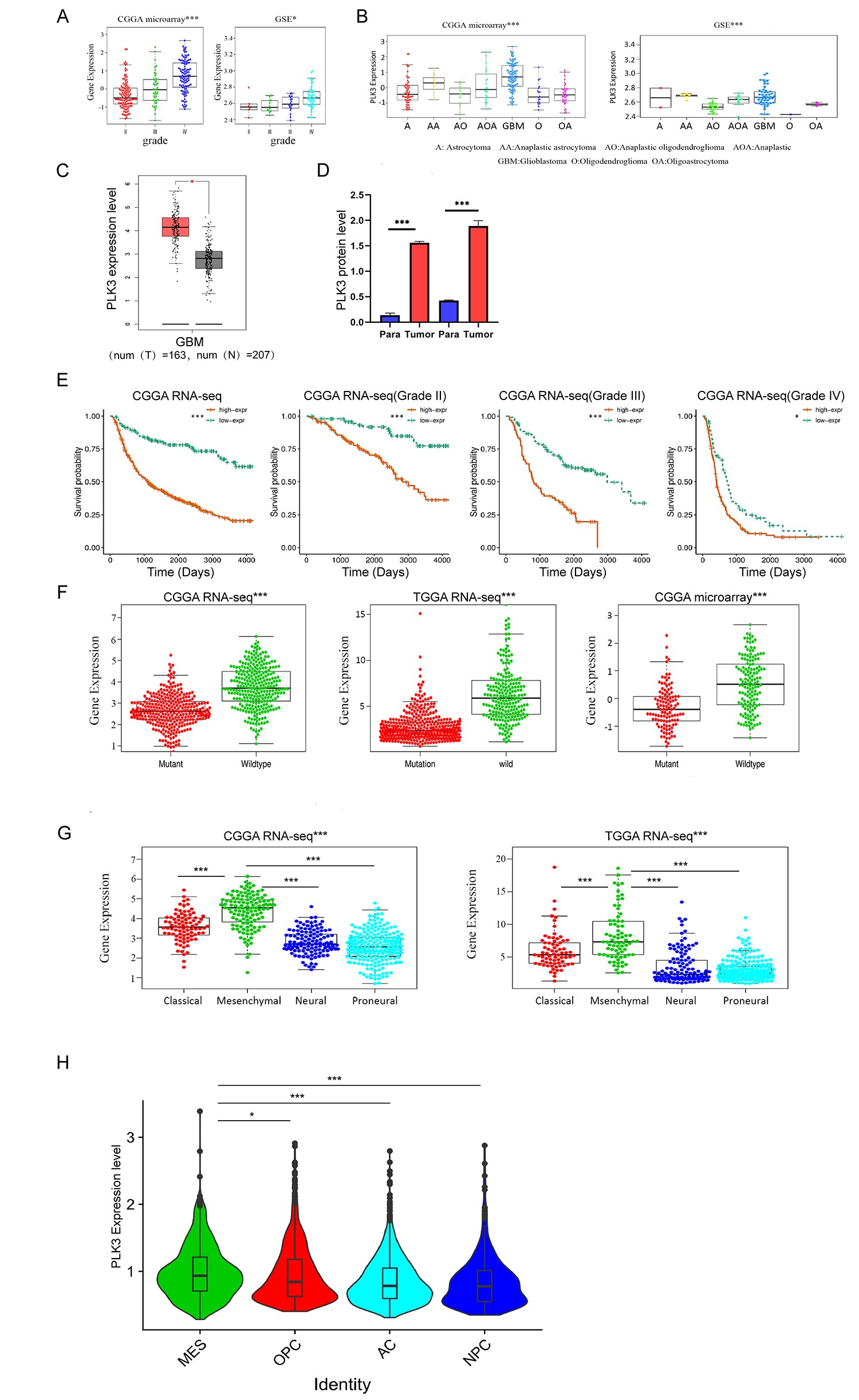

Supplement: Supplementary Fig. S1 [file OncolRes-33-50794-s001.tif]

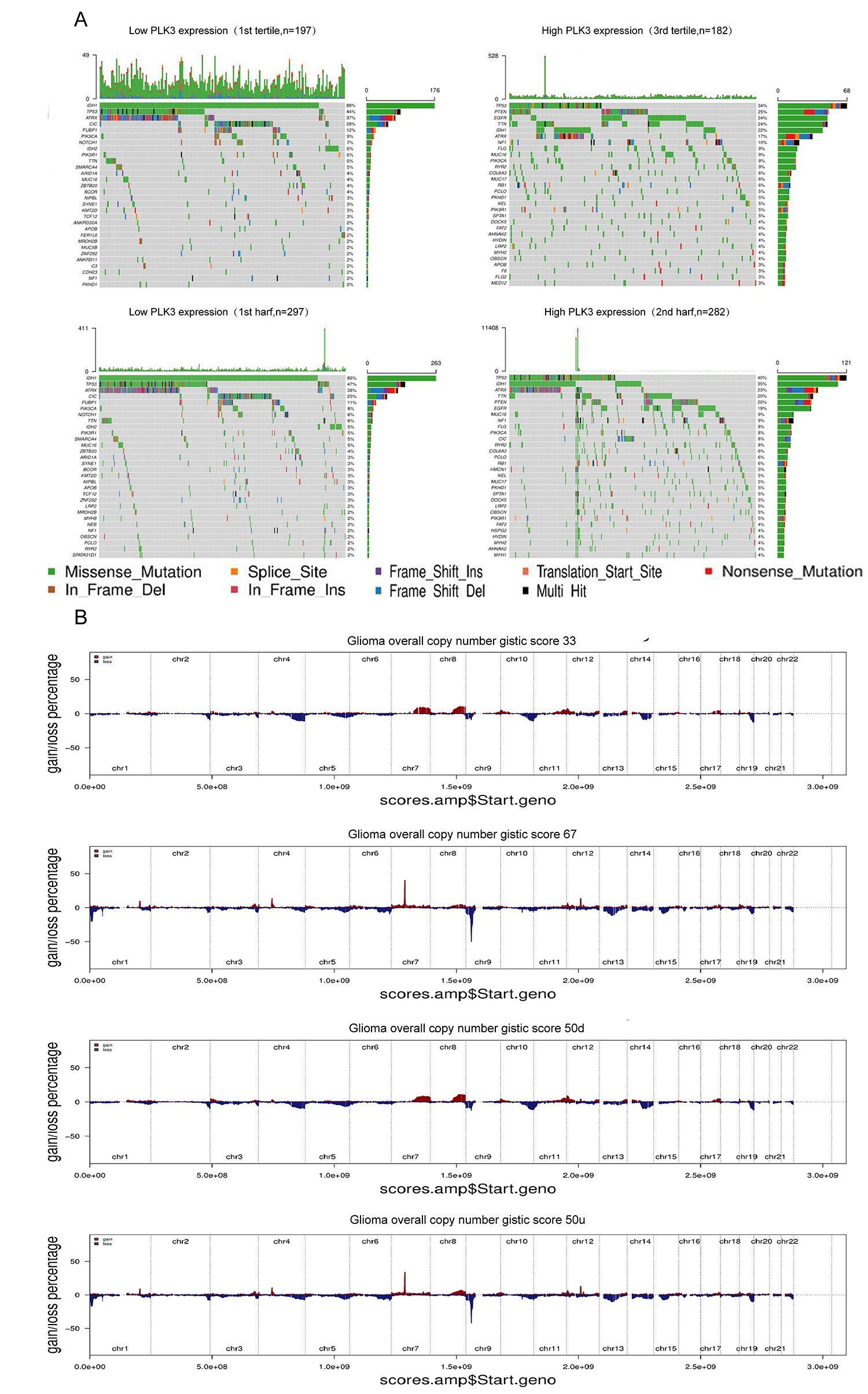

Supplement: Supplementary Fig. S2 [file OncolRes-33-50794-s002.tif]

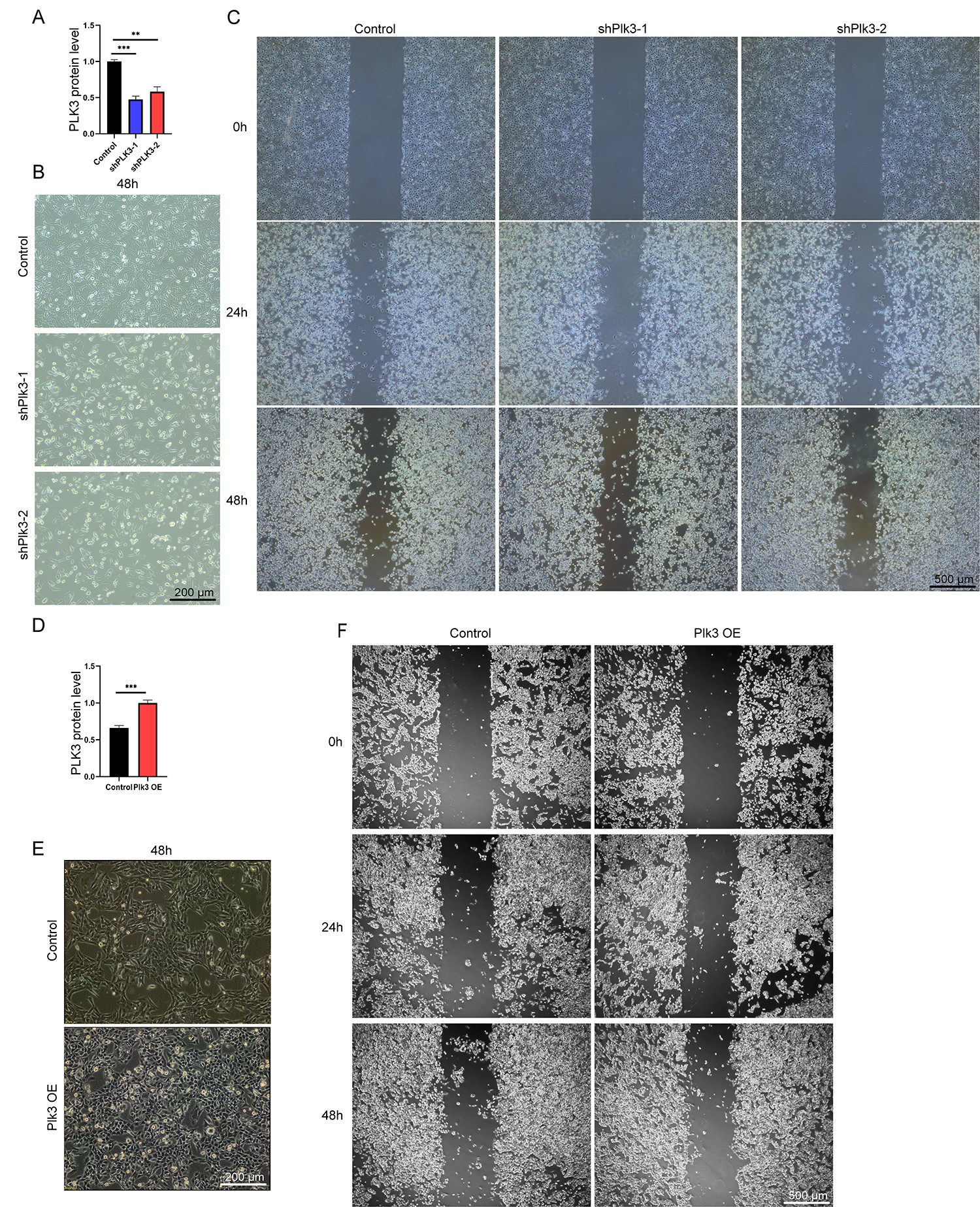

Supplement: Supplementary Fig. S3 [file OncolRes-33-50794-s003.tif]

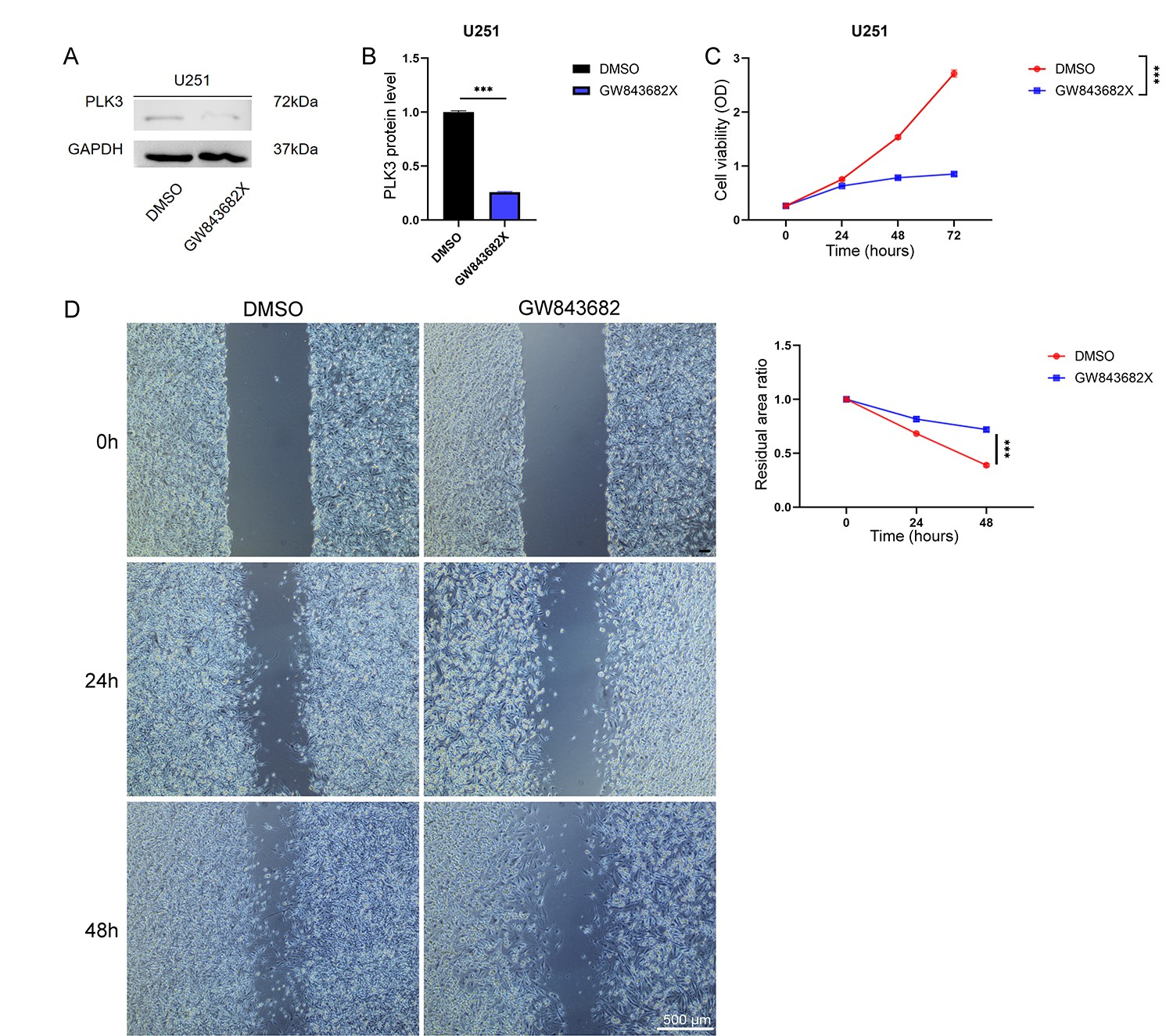

Supplement: Supplementary Fig. S4 [file OncolRes-33-50794-s004.tif]

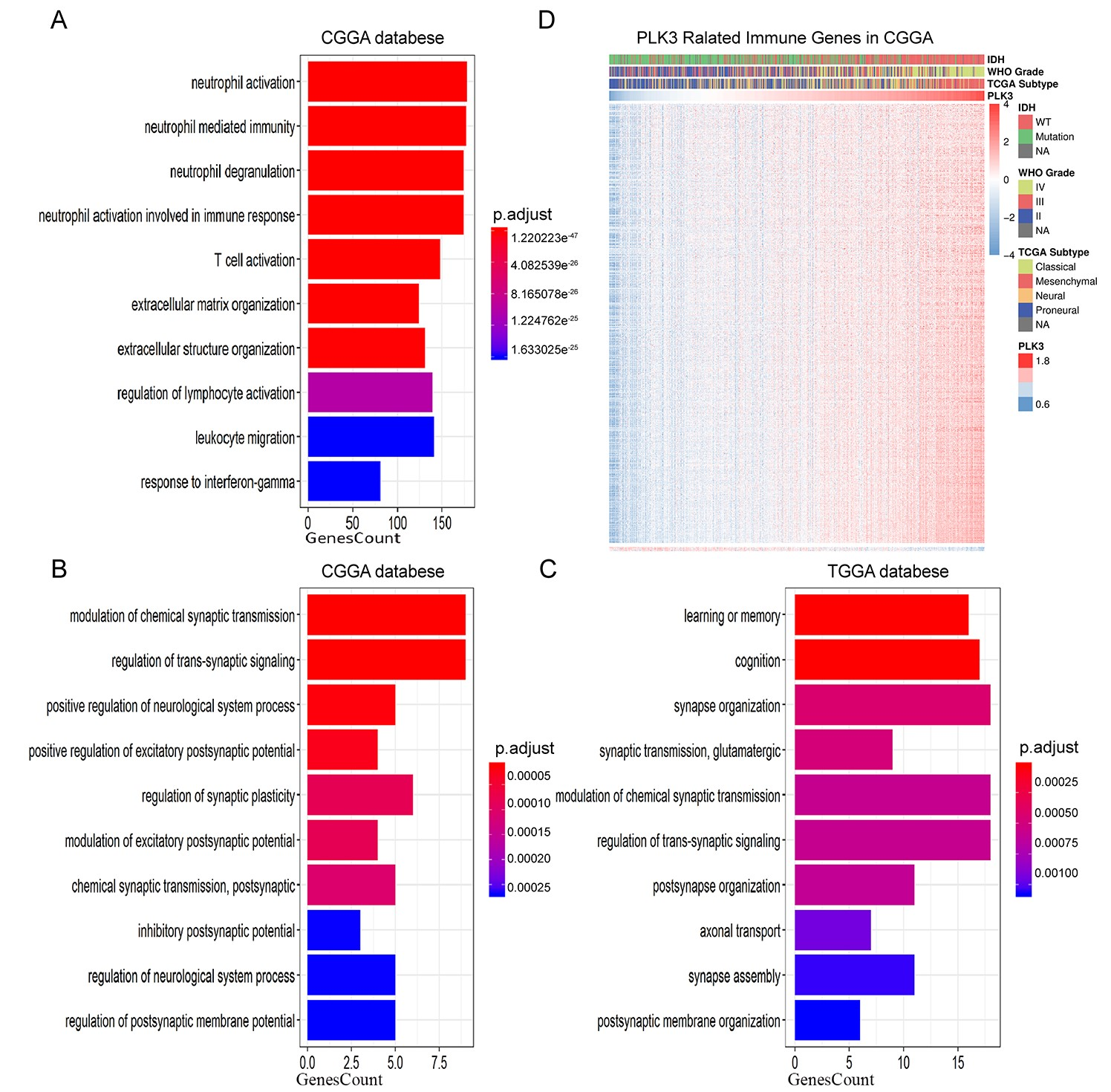

Supplement: Supplementary Fig. S5 [file OncolRes-33-50794-s005.tif]

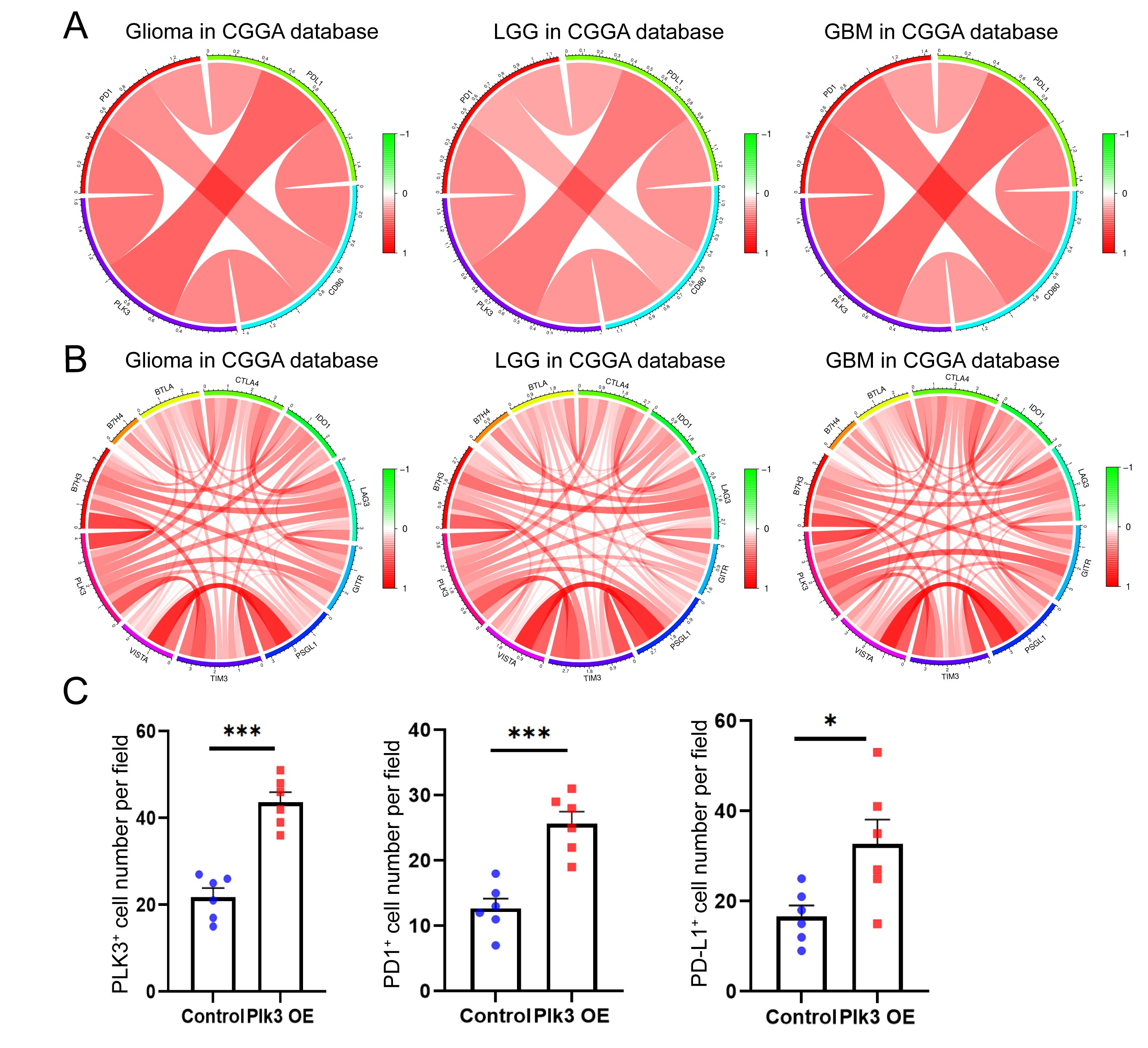

Supplement: Supplementary Fig. S6 [file OncolRes-33-50794-s006.tif]
